# Cell Wall Proteome of Wheat Grain Endosperm and Outer Layers at Two Key Stages of Early Development

**DOI:** 10.3390/ijms21010239

**Published:** 2019-12-29

**Authors:** Cherkaoui Mehdi, Lollier Virginie, Geairon Audrey, Bouder Axelle, Larré Colette, Rogniaux Hélène, Jamet Elisabeth, Guillon Fabienne, Francin-Allami Mathilde

**Affiliations:** 1 INRAE, UR BIA, F-44316 Nantes, France; mehdicherkaoui04@gmail.com (C.M.); virginie.lollier@inra.fr (L.V.); audrey.geairon@inra.fr (G.A.); axelle.bouder@inra.fr (B.A.); colette.larre@inra.fr (L.C.); helene.rogniaux@inra.fr (R.H.); fabienne.guillon@inra.fr (G.F.); 2 Laboratoire de Recherche en Sciences Végétales, Université de Toulouse, CNRS, UPS, 31326 Castanet Tolosan, France; jamet@lrsv.ups-tlse.fr

**Keywords:** cell wall, grain, remodeling, polysaccharide, proteome, wheat

## Abstract

The cell wall is an important compartment in grain cells that fulfills both structural and functional roles. It has a dynamic structure that is constantly modified during development and in response to biotic and abiotic stresses. Non-structural cell wall proteins (CWPs) are key players in the remodeling of the cell wall during events that punctuate the plant life. Here, a subcellular and quantitative proteomic approach was carried out to identify CWPs possibly involved in changes in cell wall metabolism at two key stages of wheat grain development: the end of the cellularization step and the beginning of storage accumulation. Endosperm and outer layers of wheat grain were analyzed separately as they have different origins (maternal and seed) and functions in grains. Altogether, 734 proteins with predicted signal peptides were identified (CWPs). Functional annotation of CWPs pointed out a large number of proteins potentially involved in cell wall polysaccharide remodeling. In the grain outer layers, numerous proteins involved in cutin formation or lignin polymerization were found, while an unexpected abundance of proteins annotated as plant invertase/pectin methyl esterase inhibitors were identified in the endosperm. In addition, numerous CWPs were accumulating in the endosperm at the grain filling stage, thus revealing strong metabolic activities in the cell wall during endosperm cell differentiation, while protein accumulation was more intense at the earlier stage of development in outer layers. Altogether, our work gives important information on cell wall metabolism during early grain development in both parts of the grain, namely the endosperm and outer layers. The wheat cell wall proteome is the largest cell wall proteome of a monocot species found so far.

## 1. Introduction

The wheat grain is a caryopsis composed of an embryo separated from the starchy endosperm and aleurone cells by the scutellum. The progeny is surrounded by the maternal tissues, which are composed of several outer layers [1]. They consist of the pericarp, the testa, and the nucellar epidermis [2]. The pericarp includes epidermal cells, parenchyma cells, and cross and tube cells. Cytological and physiological features of wheat endosperm and pericarp were characterized all along the grain development [3,4,5].

At the early developmental stage, the outer layers constitute most of the grain volume [3]. By around 150 growing degree days (GDD), the layers of the outer integument are the first to degenerate, whereas endosperm cells continue to divide. The beginning of grain filling occurs around 220 GDD. The aleurone layer becomes recognizable. Endosperm cells stop dividing and the grains reach their maximum fresh mass at around 440 GDD. Concomitantly with the fusion of the pericarp with the maternal epidermis, the expanding endosperm compresses the outer layers. At grain maturity, except for the aleurone cells and embryo, all tissues degenerate, and only cell walls remain.

Cell walls play key roles during grain development. They have various compositions depending on the cell type and developmental stages. Due to their importance in nutrient transport, as well as cell size, shape, and stiffness, cell walls are essential for the determination of the final grain size and the nutritional quality of cereal grain [6,7,8]. They account for 3% of the endosperm, and around 70% of the outer layers in the mature wheat grain [9]. The cell walls are the primary contributors to cereal dietary fiber with important implications for human nutrition and disease prevention [10]. Cell walls also have important effects on cereal processes, such as milling, brewing, and breadmaking quality [11]. They are mostly composed of polysaccharides, together with a smaller amount of proteins, lipids, and phenolic compounds that participate in the cell wall metabolism.

Cell walls of developing endosperm have recently been described in detail [12,13,14,15,16,17]. Despite their important roles, both in grain physiology and from a nutritional point of view, cell walls of the outer layers of cereal grain have been poorly studied so far. Callose was the first polysaccharide to be detected during the development of wheat grain [18]. Arabinoxylans (AXs) and mixed β-glucans (MLGs) are the main polysaccharides of wheat grain, and of grasses more broadly. The remaining polysaccharides consist of cellulose, mannans, xyloglucans, and pectins [14,15,18]. MLGs are deposited early during the cellularization step and throughout the grain development. AXs are detected at the beginning of the differentiation in the endosperm cells. Spatial and temporal variations of the AX structure were observed in wheat grain. This structural heterogeneity is mainly explained by the variation in the substitution degree of AXs by arabinose, but also by ferulic acid, and to a lesser extent, p-coumaric acid, which binds to the arabinose residues [12,19]. Although less abundant than in dicot plants, cellulose was observed not only in the outer layers, but also in endosperm cells in a proportion that was not expected [20]. Recently, pectins were detected in the endosperm and the outer layers of wheat grain [14]. An impressive deposition of homogalacturonans (HGs) in the testa was reported at 250 GDD, with a decrease in their methylation degree during grain development. Rhamnogalacturonans of type I (RGI) were mainly detected in the endosperm and mostly in aleurone cells. Spatial and temporal variations of the RGI structure have been observed [14].

In addition to variability of the polysaccharide distribution and structures, the cell walls of the outer layers contain significant amounts of hydrophobic and polyphenolic polymers that compose the lignin and/or cutins. Lignification occurs earlier than previously reported in the outer layers and long before the grain reaches its final size [2]. Several cuticles are formed early in the development and persist at grain maturity. They are considered to be physically associated with the cell walls and have some overlapping functions. These cuticles are mainly composed of cutins and soluble waxes. Polysaccharides are also embedded into the cuticular layers [21]. Recently, xyloglucans were detected in the cuticle above the testa in the grain of the wild grass *Brachypodium distachyon* [22].

As described above, the composition and structure of cell wall polysaccharides vary during grain development and according to grain tissue to answer to the physiological needs of plants. Cell wall polysaccharide modifications imply numerous modifying enzymes, among which, glycosyl hydrolases (GHs) act on polysaccharides and oxidoreductases. They are mainly located in the cell wall and participate in the cell elongation, cuticle deposition, and cell wall structural variation that occurs during grain development. They may be transported by vesicles from the cytoplasm to the extracellular compartment [23]. The identification and functional understanding of these protein actors are essential to gain access to cell wall dynamics. To date, only a few experiments have been reported on grass cell wall proteomes: *Brachypodium distachyon* [24,25,26], *Saccharum* spp (sugarcane) [27,28,29], *Oryza sativa* (rice) [30,31,32,33], and *Triticum aestivum* (wheat) [34]. Transcriptomic data on the developing wheat grain allowed for the identification of GH genes [15], and recently, we reported the first data on the cell wall proteome of endosperm and the outer layers of wheat grain at a given developmental stage [34]. More than 600 cell wall proteins (CWPs) were identified, half of which were present in both tissues, suggesting common and specific tissue remodeling activities.

In this study, we investigated the cell wall proteome targeting both the endosperm and outer layers of the wheat grain at two key developmental stages in order to report on the cell wall dynamics during grain development. We identified proteins annotated as CWPs that are potentially involved in cell wall polysaccharide remodeling and cell wall assembly. A quantitative analysis allowed for describing the cell wall proteome content during the grain developmental stage for two tissues: the endosperm and the outer layers.

## 2. Results

### 2.1. Developing Grain Stages

**Figure 1:**
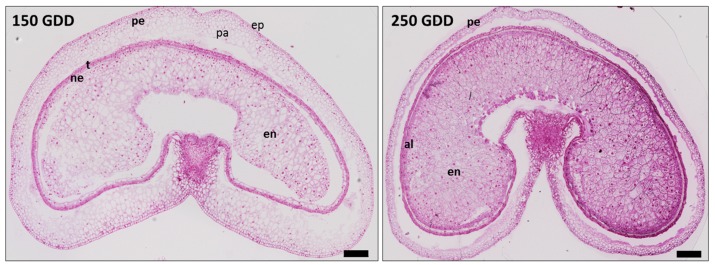
Tissue differentiation and development of wheat grain. Wheat grain cross-sections at (**left**) 150 and (**right**) 250 growing degree days (GDD) using bright-field micrographs of toluidine blue-stained sections. pe: pericarp, ep: external pericarp, pa: pericarp parenchyma, ne: nucellus epidermis, t: testa, al: aleurone layer, en: endosperm. Scale bars: 250 μm.

The aim of this work was to identify CWPs in order to better understand the cell wall dynamics during the development of wheat grains. For this, the experiments were conducted from grains harvested at two developmental stages. The first one corresponded to an early stage of development (150 GDD, Figure 1), where the cellularization step was almost achieved but endosperm cells continued to divide. Aleurone and transfer cells were not yet differentiated. The outer layers derived from maternal tissues were already well differentiated. The outer layers were composed of the pericarp, the testa, and the nucellus epidermis (Figure 1).

The second developmental stage corresponded to the beginning of the accumulation of storage products. At this stage, around 250 GDD, endosperm was well differentiated. Aleurone and transfer cells were easily detected. In addition, the outer tissues progressively began to degenerate (Figure 1).

### 2.2. Protein Extraction and Proteomic Analysis

**Figure 2:**
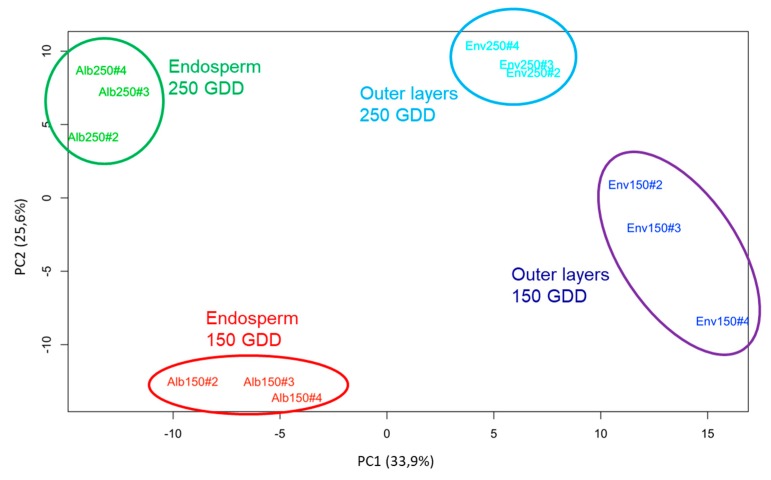
Principal component analysis (PCA) performed using the relative amounts of cell wall proteins (CWPs) for both parts of the wheat grain and at the two developmental stages (150 and 250 GDD). Individual maps of the biological replicates (red: endosperm samples at 150 GDD, green: endosperm samples at 250 GDD, dark blue: outer layers samples at 150 GDD, light blue: endosperm samples at 250 GDD) along principal components 1 and 2.

For our analysis, the endosperm and outer layers were manually separated from grains at 150 and 250 GDD. Then proteins were extracted from three independent biological replicates per condition. Our procedure led to an enrichment of CWPs in the extracts. The electrophoretic profiles of replicates were similar and large number of bands was observed between 10 and 120 kDa (Figure S1). For each replicate, around 0.5 mg and 0.8 mg of proteins were obtained from 500 mg of fresh mass of endosperm and outer layers materials, respectively. Fifty micrograms of proteins were separated using 1DE (one dimension electrophoresis) and each lane was cut into five slices prior to being trypsin-digested and analyzed using LC-MS/MS. Only proteins identified as having at least two specific peptides were conserved. Interrogation of the UniProt databank restricted to *T. aestivum* led to the identification of 2454 proteins (Table S2). The principal component analysis (PCA) indicated a good clustering of biological replicates and a significant discrimination between the four experimental conditions (Figure 2). The first two components explained 59.5% of the total variance. The different replicates clustered quite separately according to the tissue along the first principal component, and the development stage along the second principal component.

The predicted subcellular localization and functional annotation were achieved using the *ProtAnnDB* pipeline. A dataset of 734 total CWPs was selected, as described in Material and Methods (Table S2), which represents about 30% of all the 2454 identified proteins. In the outer layers, the CWP enrichment was 39%, while in the endosperm it was 22%. CWPs identified from the developing grains of *B. distachyon* represented around 40% of the total number of identified proteins, i.e., in the same range as what was obtained for the outer layers of the wheat grain [25]. Given that the complete genome annotation of wheat has currently not been achieved, a particularly large number of proteins were annotated as “uncharacterized protein”. By searching predicted functional domains using the *ProtAnnDB* pipeline, the annotation of our data was refined, leading to the functional annotation of a significant number of additional proteins, thus drastically decreasing the amount of “uncharacterized proteins”.

In addition, a statistical analysis of the quantitative data obtained for CWPs was performed in order to consider the variation of CWP abundance between the two developmental stages and the two parts of the grain. The relative quantification of proteins was performed from the signal intensity of their respective peptides in mass spectroscopy (MS), using a label-free and non-targeted approach. Quantification was achieved from a minimum of two peptides per protein. A total of 243 proteins was shown to present variation in their abundance according to parts of the grain and/or developmental stages (Table S3).

### 2.3. Validation of Cell Wall Localization for Three of the Identified Proteins

**Figure 3:**
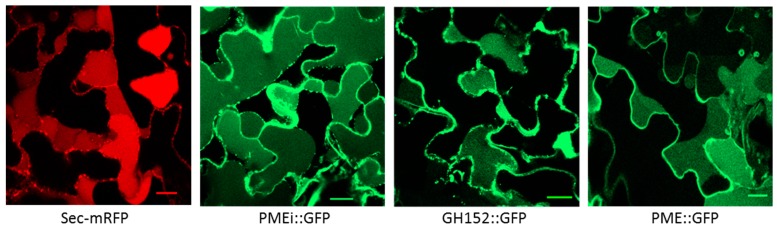
Subcellular localization of a PMEI (A0A1D6BK49), a GH152 (A0A1D6DD47), and a PME (A0A1D5V0T8) fused to a green fluorescent protein (GFP) in plasmolyzed *N. tabacum* leaf epidermal cells. Confocal images of plasmolyzed cells expressing the apoplastic marker Sec-mRFP, PMEI::GFP, GH152::GFP, and PME::GFP in leaf epidermal cells 2–3 days after agroinfiltration. Scale bars: 20 μm.

In order to validate our proteomic analysis and bioinformatics treatment, we decided to check the cell wall localization for three randomly selected CWPs. These proteins were respectively annotated as a pectin methylesterase (PME), a PME inhibitor (PMEI), and a glycoside hydrolase belonging to family 152 (GH152). We therefore generated C-terminal green fluorescent protein (GFP) fusions under the control of the cauliflower mosaic virus (CaMV) 35S constitutive promoter using an appropriate binary vector. The sequences used for the constructs encoded the full length CWPs, including their signal peptides. The constructs were transiently expressed in *N. tabacum* leaves after *A. tumefaciens* infiltration. The subcellular localization of the fusion proteins was analyzed using confocal laser scanning microscopy. The fluorescence of the protein fusions PME::GFP, GH152::GFP, and PMEI::GFP was detected at the periphery of the cells; this was consistent with their localization in the cell wall. A plasmolysis of the infiltrated leaf pieces was performed to separate the plasma membranes from the cell walls. The results indicated that the three fusion proteins were present in the cell wall and in the apoplastic compartment, as observed with the apoplastic marker Sec-mRFP (Figure 3).

### 2.4. Distribution of the CWPs in Functional Classes

**Figure 4:**
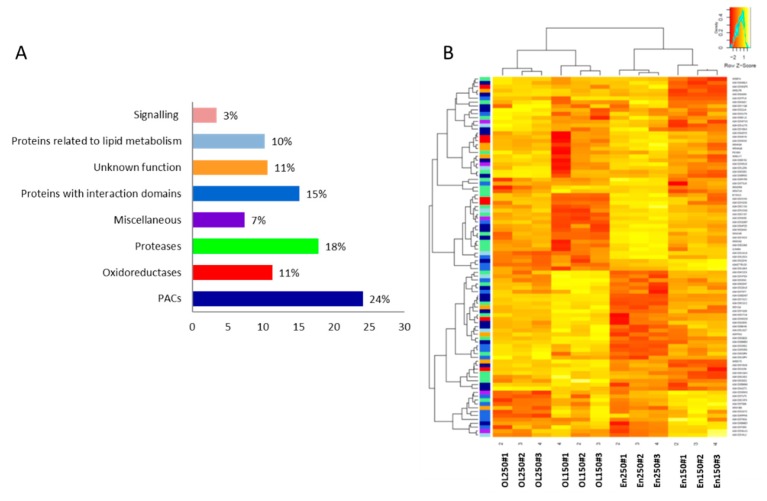
Distribution of CWPs and variation of their abundance according to the different conditions. (**A**) Distribution of CWPs into functional classes according to their predicted functions. PACs: proteins acting on cell wall polysaccharides. (**B**) Heatmap of the relative amounts of CWPs identified in the four conditions (in the endosperm (EN), in the outer layers (OL), and at 150 and 250 GDD).

The CWPs are distributed into eight of the nine functional classes, as defined by the *ProtAnnDB* tool [35] (Figure 4A). No protein was annotated as a structural protein that has been currently observed in cell wall proteomes of monocots [36]. The most effective functional class was that of “proteins acting on cell wall polysaccharides” (PACs) with around a quarter of the CWPs. Inside this functional class, we mainly found GHs, which was consistent with previous cell wall proteome studies [25,26,28,37] (Table S2). PACs also contained expansins, carbohydrate esterases, and to a lesser extent, pectate lyases, PNGases (*N*-glycosidases), and COBRA-like proteins. Altogether, 136 GHs were identified. They belong to 21 different GH families (http://www.cazy.org/). They are thought to play various functions in cell expansion, signaling, plant defense, and the hydrolysis of glycans from glycoproteins and polysaccharides [38]. GHs were fairly distributed between the developmental stages and grain parts. Among them, the best-represented families were GH3 and GH17, as previously found in *B. distachyon* grains [24]. The classes of “proteases” and “proteins with interacting domains” were also well represented. The functional class of “signaling proteins” accounted for 4% of the total CWPs, as currently found in other cell wall proteome of monocots (3.4% for overall cell wall proteomes of monocots [39]; 6% if only the cell wall proteome of *B. distachyon* grains is considered [25]). Among the proteins of yet unknown function, DUF642 proteins were found as in all the previously studied cell wall proteomes (see *WallProtDB*).

### 2.5. Distribution of CWPs and Their Abundance Variation According to Tissue

**Figure 5:**
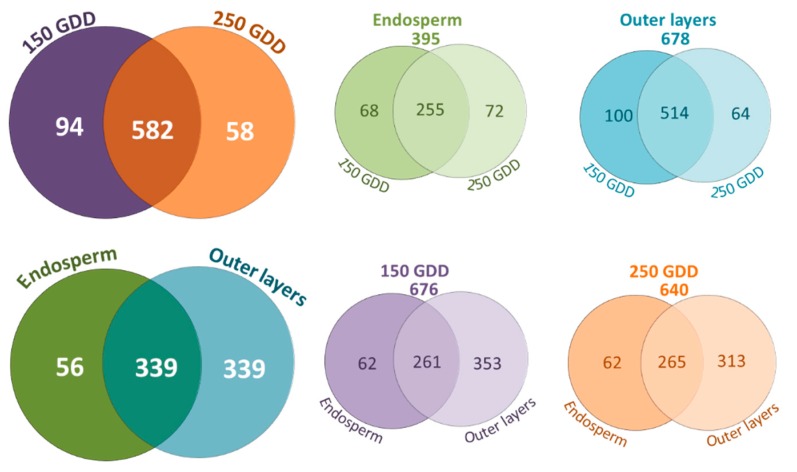
Venn diagrams showing the distribution of CWPs according to the wheat developmental stages and grain parts.

Among the CWPs, almost two times more proteins were identified in the outer layers compared to the endosperm (Figure 5). More than 300 CWPs were shared between the two parts of the grain.

Among the CWPs identified in the endosperm, few of them were specific to this tissue. Several of them were annotated as proteins with leucine-rich repeats (LRR proteins), class III peroxidases, multicopper oxidases, or plant invertases/PME inhibitors, and others corresponded to the COBRA-like, DUF296, DUF239, and antimicrobial peptide (AMP) protein families. No GH family was found exclusively in the endosperm. In contrast, the outer layers accounted for numerous specific CWPs (Figure 5, Table S2) with proteins belonging to families of pectate lyases, acetyl xylan esterases (AXEs), GH10, GH13, GH32, laccases, and some DUF proteins (DUF642, 1929, 568, and 760).

Although numerous protein families were identified, both in the endosperm and outer layers, some of them differed by the number of their members between these two tissues. Some protein families were best represented in one of these two grain parts. This was the case for the fasciclin-like arabinogalactan proteins (FLAs), GDSL lipases/esterases, GH31, GH35, class III peroxidases, purple acid phosphatases (PAPs), and pectin acetylesterases (PAEs), which were more represented in the outer layers. Due to the relatively low number of CWPs identified in the endosperm compared to the outer layers, it was more difficult to reveal the protein families best represented in this tissue. However, our results clearly indicated that proteins with a protease-associated (PA) domain, α-amylase inhibitors, GH28, GH29, and plant invertases/PME inhibitors were well represented in the endosperm (Table S2).

Considering the developmental stages, only a third of the CWPs were shared by the endosperm and outer layers (Figure 5). This was partly due to the big difference in the numbers of CWPs identified in the endosperm and outer layers. The classes of “oxidoreductases,” “proteins related to the lipid metabolism,” and “proteins possibly involved in signaling proteins” were more numerous in the outer layers compared to the endosperm (Figure S2). Inversely, the class of “proteins with interacting domains” was best represented in the endosperm, with a significant proportion of CWPs annotated as plant invertase/PME inhibitors (Figure S2, Table S2).

From a statistical point of view, variations of the CWPs’ abundance were observed between the endosperm and outer layers at a given developmental stage (150 GDD or 250 GDD) (Table S3, Figure S2). Among the CWPs shared by the endosperm and outer layers at 150 GDD, 60 were accumulated at a higher level in the outer layers, and only 28 in the endosperm. At 250 GDD, the numbers of CWPs accumulated at a high level in the outer layers and endosperm were closer (57 CWPs in outer layers versus 47 in endosperm), revealing a strong cell wall dynamic in both parts of the grain. More dirigent proteins, oxidoreductases, and thaumatins were accumulated at a higher level in the outer layers than in the endosperm, especially at 150 GDD. Some GHs (GH1, GH17, and GH79) were also accumulated in the outer layers, whatever the developmental stage. In the endosperm, plant invertases/PME inhibitors were found to have greater levels of accumulation compared to the outer layers, as well as some protease inhibitors and GH3.

### 2.6. Distribution of CWPs and Their Abundance Variation According to the Developmental Stage

Few differences were noticed between the total number of CWPs identified at 150 GDD and 250 GDD (676 vs. 640 CWPs) (Figure 5, Table S2). A large majority of them were shared by the two stages. Only a few differences were observed for each protein family between the two developmental stages. However, a slightly higher number of expansins, lipid transfert proteins (LTPs), invertase/PME inhibitors, class III peroxidases, and plastocyanins was noticed at 150 GDD compared to 250 GDD. On the other side, arabinofuranosidases (GH51 and GH146), proteins with predicted protease-associated (PA) or cupin domains, and some proteases (subtilases) were slightly more represented at 250 GDD compared to 150 GDD. Whereas the GH10, DUF760, and DUF296 families were only identified at 150 GDD, lectins and COBRA proteins were only found at 250 GDD (Table S2).

Among the CWPs identified at 150 GDD, around one hundred were specific to this stage, with more proteins identified specifically in the outer layers compared to those identified in the endosperm (Figure 5, Table S2). While some invertase/PME inhibitors were specific to the endosperm at 150 GDD, several AXEs, class III peroxidases, and DUF538 proteins were specifically found in the outer layers at this earlier developmental stage. At 250 GDD, we identified 58 CWPs specific to this stage, with twice as many identified proteins in the outer layers compared to the endosperm, two arabinofuranosidases and two subtilases among them (Table S2).

Considering the endosperm, more than twice as many CWPs were significantly accumulated at higher level at 250 GDD compared to 150 GDD (52 vs. 21). The opposite was observed in the outer layers (Table S3, Figure S3). This could reflect an intensive cell wall metabolism in the outer layers at 150 GDD, whereas the cell wall metabolism in the endosperm seemed to be more dynamic at 250 GDD. In this way, numerous proteases, but also FLAs, leucine rich repeat-containing proteins (LRR-proteins), and GH9 were accumulated at 250 GDD in the endosperm. Inversely, in the outer layers, proteins annotated as AXEs, GDSL lipases/esterases, or proteases were particularly abundant at 150 GDD (Table S3).

## 3. Discussion

In this work, we investigated the cell wall proteome of two distinct parts of the wheat grain. The outer layers and endosperm are composed of distinct cell layers that have different functions in grain. Thus, the cell wall structures are very different and we were expecting to obtain distinct CWP profiles between these two parts of grain. Two early developmental stages, 150 GDD (end of cellularization step) and 250 GDD (differentiation step and beginning of storage accumulation) were considered since clear differences at the histological and cell levels (Figure 1) and in the cell wall composition have been described between these two stages [12,14,17]. Although numerous cell wall events occur after 250 GDD, the accumulation of storage proteins, such as glutenins and gliadins in wheat endosperm, makes wall preparation and proteomic analysis more difficult. Indeed, due to the amount of storage proteins being particularly high from 300 GDD, the detection of CWPs, which are by weight minor cell wall components, would be very hard.

Although the outer layers can be easily detached from the endosperm of early harvested wheat via manual dissection [40], we cannot totally exclude protein cross-contaminations between these two tissues. However, the PCA indicated a strong discrimination between the endosperm and outer layers samples collected at 150 or 250 GDD, as well as a low variability between replicates (Figure 2). Because most of the polysaccharides were deposited both in the endosperm and outer layers, and since several cell wall remodeling events occurred in both grain tissues, numerous CWPs were expected to be identified in both of them. Forty-six percent of the identified CWPs were indeed found in both parts of grain.

To analyze the cell wall proteome, we performed cell wall enrichment and the proteins were extracted with CaCl_2_ and LiCl buffers using a protocol successfully used on different plant species and tissues [25,26,28,29,37,41,42]. In this work, we identified a total of 2454 proteins, 734 of them being predicted as secreted proteins present in the cell wall or the apoplast, and called CWPs. CWPs were more numerous in the outer layers compared to the endosperm. The outer layers contained several cell layers, whereas endosperm tissues only contained starchy endosperm and aleurone cells. This could explain why more CWPs were identified in the outer layers, reflecting the presence of more different cell types. In addition, the outer layers contained more cell walls than the endosperm, leading to a higher protein amount. Also, we cannot exclude a better extractability of proteins from the outer layers than from endosperm. Conversely, almost the same number of CWPs were identified at both developmental stages of grains, with around 90% being shared proteins. These results, reinforced by the PCA data, suggest that in our experiments, the tissue effect was stronger than the developmental stage effect. However, many cell wall metabolism events differed from 150 to 250 GDD, such as the aleurone cell differentiation or the start of the pericarp degeneration, which may involve different CWPs [3,5]. Additionally, these distinct metabolic events were certainly reflected by differences in protein accumulation (Table S3). Many CWPs were more abundant in the endosperm at 250 GDD (in particular expansins, GH9, or GH28), while accumulation of a large number of CWPs was observed at 150 GDD in the outer layers (with numerous GDSL lipases/esterases, or oxidoreductases, such as class III peroxidases) (Table S3, Figure S3).

The distribution of wheat grain CWPs into functional classes slightly differed from those of the other cell wall proteomes. It appeared that the cell wall proteomes of grass grains (wheat and *B. distachyon*) contained more proteases and less oxido-reductases than the cell wall proteomes already described (Figure 4, [24,25,26,27,28,29,30,31,32,33,34,35,36,37,38,39,40,41,42,43,44,45,46]). The cell wall proteome of wheat grain also stood out as having numerous proteins with predicted interaction domains compared to other cell wall proteomes, including that of the *B. distachyon* grain [24,25]. Among proteins with interaction domains, more than 50 CWPs belonged to the superfamily of the “plant invertase/PME inhibitors”. Although found in all conditions, these proteins were more represented in the endosperm, and at 150 GDD. The transient expression of a fusion protein plant invertase/PME inhibitor::GFP in tobacco leaves confirmed its presence in the cell wall and in the apoplast (Figure 3). Wheat has a complex genome, with six copies of each chromosome and many nearly identical sequences scattered throughout, which could account for the large number of protein isoforms. As a matter of fact, many of the identified plant invertase/PME inhibitors present a strong homology. In wheat grain, there is a considerable number of plant invertase/PME inhibitors compared to other plant cell wall proteomes. In the cell wall proteomes of *A. thaliana* and *B. distachyon* (compilated data from *WallProtDB*), only 12 and 7 plant invertase/PME inhibitors were identified, respectively. In *B. distachyon*, they were all found in the developing grain [25]. In rice, several of the 54 predicted invertase/PME inhibitors are expressed specifically in developing endosperm [44]. PMEIs inhibit the demethylesterification of HGs by PMEs [45]. In wheat, PMEIs were suggested to play a role in plant defense mechanisms by decreasing the cell wall porosity and/or by conferring resistance against several pathogens [46,47]. Recently, two invertase inhibitor genes were identified in sugarcane, one of them encoding a protein (ShINH1) localized in the apoplast of the root, flower, stalk, and leaf. Heterologous expression of *ShINH1* confirmed its possible function as an invertase inhibitor [48]. In the same way, an apoplastic invertase inhibitor was shown to affect seed mass in soybean by regulating extracellular invertase during seed maturation [49]. Invertase inhibitor ZM-INVINH1 has been reported to bind cell wall invertase during grain development in maize [50]. Invertases belong to the GH32, GH68, and GH100 families. In our study, five GH32 that were predicted to be secreted were only found in the outer layers, which is consistent with a preferential presence of such proteins in the endosperm. On the other hand, the high amount of the plant invertase/PME inhibitors raises the possibility that they could regulate other carbohydrate metabolizing enzymes, as previously suggested [44].

Additional protein families were also well represented in the endosperm, such as GHs belonging to the GH9, GH28, and GH38 families, respectively annotated as endo-β-1,4-glucanases, polygalacturonases, and α-mannosidases. GH28 proteins were potentially active on HGs. Although present at the both developmental stages, some were more abundant at 250 GDD in the endosperm. It was already surprising to identify numerous GH28s in the cell wall proteome of the *B. distachyon* grain, at three developmental stages (9, 13, and 19 days after flowering), pectin also being poorly abundant in this plant species [25]. Although cell walls of grasses contain low level of pectins (HGs and rhamnogalacturonans), the presence of such enzymes is consistent with the remodeling of early synthesized pectins in the developing grain [14,17]. Two putative α-mannosidases belonging to the GH38 family were previously identified in the developing grain of *B. distachyon*, but no data are available concerning their fine localization [25]. Many plant α-mannosidases have been isolated and characterized from seeds and fruits. More generally located in the Golgi or endoplasmic reticulum, they are known to be involved in the maturation of *N*-glycans, and they appear to play a critical role in plant development [51,52]. Finally, the quantitative data indicated that GH9 were more abundant at 250 GDD than at 150 GDD in the endosperm. These enzymes are usually able to hydrolyze artificial soluble cellulose derivatives, such as carboxymethyl cellulose (CMC) or hydroxyethyl cellulose (HEC), and biochemical analyses have revealed their specificity for different substrates in vitro [53]. More generally, a large proportion of the endosperm CWPs were more abundant at 250 GDD than at 150 GDD, suggesting a more intensive cell wall metabolic activity in this tissue at this developmental stage of grain. This could be explained by the differentiation of the aleurone cells in addition to the expansion of the starchy endosperm cells at the beginning of storage accumulation.

Our work revealed significantly different cell wall proteomes between the endosperm and outer layers of the wheat grain, which could be largely explained by the differences of the cell wall structures between these two parts of grain. Thus, numerous protein families, highly represented in the cell wall proteome of outer layers, are known to act not only on cell wall polysaccharides but also on hydroxycinnamic acids, lignin, or cuticle formation, the last two being characteristic of the outer layers. This was the case with the GDSL lipases/esterases being highly numerous at 150 GDD. These proteins are members of a large family that display numerous functions, such as cutin synthase, cutin hydrolase, deacetylation of polysaccharides, polysaccharide hydrolysis, fucosidase, and esterification of secondary metabolites [54]. They certainly collaborate to implement the cuticle during grain development, including the association of cutin with polysaccharides and the rearrangement of cutin and polysaccharides [54]. In wheat, cuticles were observed early during grain development [14], which is consistent with the presence of GDSL lipases/esterases at 150 GDD in outer layers. The accumulation of lipid transfer proteins (LTPs) in the outer layer matches with their role in the synthesis of cuticular waxes [21,55]. However, although less numerous, their presence in the endosperm suggest another function, such as antimicrobial defense or signaling during pathogen attacks [55]. Beside the cuticle, other non-polysaccharidic components are present in cell walls, such as lignin or hydroxycinnamic acids. Feruloylated AXs and lignins were indeed detected early in developing grain [14]. Current evidence suggests that two enzyme families, class III peroxidases and laccases, are involved in lignin polymerization [56]. These proteins were identified in the outer layers of developing grain in wheat. One laccase was identified only in the outer layers and numerous class III peroxidases were found mainly in the outer layers at 150 GDD with several of them more abundant at 150 GDD than at 250 GDD. Moreover, class III peroxidases could also be involved in AX crosslinking by ferulic acid, as previously shown in maize [57].

The outer layers and endosperm exhibit distinct functions that might explain differences of the cell wall proteomes. For instance, we found eight members of the GH19 family with four of them only identified in outer layers. In addition, the GH19 found in the two parts of the grain were always more abundant in the outer layers. These proteins are involved in the biotic stress response and they are known to be defense weapons against fungi by exhibiting chitinase activity [58]. These findings correlate with the protective role of outer layers throughout grain development. Among the protein families of the PAC class, many have a higher population in the outer layers. This is the case for the DUF642 proteins, which were identified only in the outer layers at both developmental stages. The DUF642 proteins belong to a well-conserved family. They were shown to be involved in plant development [59]. They may modulate the activity of PMEs, which are more abundant in the outer layers than in the endosperm, suggesting that their function could be related to the regulation of HG modifications by interacting with the catalytic domain of PME. Their interaction with cellulose was also demonstrated in vitro but no functional studies yet confirm these interactions [60]. Pectin acetylesterases (PAEs) and AXEs were almost only found in outer layers. Acetylated AXs were detected in young wheat grain (245 GDD), but not at a mature stage (700 GDD) [61]. AXEs could participate in the deacetylation of AXs during the grain development. The deacetylation of AXs could improve the rigidity of the cell wall, as deacetylation of xylans facilitates their bonding to cellulose [62].

Other protein families, even though not specific to one developmental stage, are best represented at 150 GDD and in the outer layers, particularly the expansin, DUF538 proteins, and plastocyanins. In wheat, expansin genes were reported to be expressed at a high level during early grain expansion, with a peak of *TaExp6* transcript accumulation in the pericarp at 141 GDD, and a higher level of expression in the pericarp and endosperm at 187 GDD [7]. These results are consistent with the identification of more expansins at the cellularization step, and the presence in both the endosperm and outer layers. Moreover, the role of expansins in grain is reinforced by a recent study suggesting that expansin genes could be specifically involved in grain enlargement and may control seed growth in sunflower at the early phases of development [63]. As in wheat, several DUF538 were identified in the developing grain of *B. distachyon* [25]. Several functions have been suggested for proteins containing this domain of yet unknown function, such as their involvement in the photosynthetic system of plants by degrading chlorophyll molecules to antioxidant compounds under stress induction [64,65]. Initially characterized as chloroplastic proteins, some DUF538 proteins were shown to be in other cell compartments. Recently, a maize DUF538 protein, Glossy6, was demonstrated to be involved in cuticular wax accumulation and drought tolerance [66]. Proteins annotated as plastocyanin-like proteins, also named plantacyanins, were also preferentially found at 150 GDD and in the outer layers. They are blue copper-binding proteins belonging to the oxidoreductase class, identified in numerous cell wall proteomes, such as in stems, leaves, and the developing grain of *B. distachyon* [24,26]. Recently it was shown that the blue copper proteins from the phytocyanin family were involved in the control of panicle branching and grain weight in rice through their regulation by the microRNA miR408 [67].

Proteases are well represented in the early developmental stages, and especially at 250 GDD. Endoprotease activities were characterized in developing wheat grain [68]. Serine protease activities were characterized at early stages of grain development, with metalloproteases and Asp proteases appearing later. We identified proteases belonging to the Asp-protease, Cys–protease, or serine carboxypeptidase families, both in outer layers and the endosperm. In the endosperm, several of them were more abundant at 250 GDD than at 150 GDD, suggesting stronger metabolic activities at 250 GDD in the endosperm cell wall. Endoproteases could participate in cell wall dynamics through the maturation of cell wall remodeling enzymes, their degradation, or the release of signaling peptides. In the case of subtilases, forming a large Ser protease family, it was suggested they were involved in the regulation of PMEs in *Arabidopsis* seeds [69,70]. A subtilase specifically found in the endosperm of *Medicago truncatula* or *Pisum sativum* seeds during development was shown to influence seed weight [71]. It was also suggested that endoproteases may be involved in the degeneration of the pericarp from the differentiation stage to the grain maturation, whereas in the endosperm, they may be accumulated for protein metabolism (processing or turn-over) or further degradation of storage proteins [68].

## 4. Material and Methods

### 4.1. Plant Material

*Triticum aestivum* (cv Recital) was grown under natural day-length conditions in a greenhouse (INRA Le Rheu, France). Two months of vernalization were applied in a growth chamber at 8 °C before the wheat seedlings were transplanted into individual pots containing a standard potting mixture (peat RHP15 Klassman, K Klassman France, Bourgoin Jallieu, France). An Osmocote (R) Exact Tablet containing nitrogen (15%), phosphate (9%), potassium hydroxide (9%), and magnesium (3%) (Scotts International B. V., Waardenburg, The Netherlands) was added. The plants were watered daily. To harvest grains at defined developmental stages, individual ears were tagged at flowering. The temperature was recorded every day in order to follow the plant development on the basis of the cumulated temperature in growing Celsius degrees days (GDD). Plants were harvested at 150 and 250 GDD after flowering. Grains were manually dissected to separate the outer layers and endosperm and immediately stored at −80 °C.

*Nicotiana tabacum* plants were grown under a 16-h light/8-h dark photoperiod at a temperature of 24 °C day/18 °C night for five weeks in a growth chamber prior to *Agrobacterium tumefaciens* infiltration.

### 4.2. Cell Wall Enrichment and Protein Extraction

Cell wall fractionation and CWPs extraction were performed as previously described [34]. Briefly, grain samples (500 mg) were ground and centrifuged in increasing sucrose concentrations to isolate cell walls. Proteins were extracted via successive washes in a 5 mM acetate buffer at pH 4.6, supplemented with 0.2 M CaCl_2_ or 2 M LiCl. Three biological replicates were performed for each condition (developmental stages and parts of the grain).

### 4.3. Mass Spectrometry Analysis

Proteins (50 μg) were briefly separated in precast gels in order to reduce the mix complexity. Each track was cut into five fragments, which were reduced using dithiothreitol (DTT), alkylated by iodoacetamide and trypsinolyzed as previously described [34], prior to mass spectrometry (MS) analysis.

The trypsin-digested samples (6 μL) were injected into the LC-MS/MS system. The MS analyses were performed using an LTQ-Orbitrap VELOS mass spectrometer (i.e., a linear ion trap quadrupole mass filter associated with an Orbitrap^TM^ analyzer, Thermo-Fisher Scientific, Bremen, Germany) coupled to a nanoscale LC system (Ultimate U3000 RSLC system, Thermo-Fisher Scientific). Chromatographic separation was performed on a reversed-phase capillary column (Acclaim^®^ PepMapTM C18 2 μm 100 A, 75 μm i.d. × 50 cm long, Thermo-Fisher Scientific, Waltham, MA, USA) at 60 °C using a linear gradient performed between the following mobile phases: (A) 97.9% water, 2% acetonitrile (ACN), 0.1% trifluoroacetic acid (TFA); and (B) 90% ACN, 0.08% formic acid. The other parameters were as described previously [72]. Analyses were performed using a typical survey method, in which full MS scans were acquired at 60,000 resolution (full width at half-maximum, FWHM) using the Orbitrap analyzer (*m*/*z* 300–2000), while the CID (collision induced dissociation) spectra for the eight most intense ions were recorded in the LTQ.

### 4.4. Bioinformatics Treatment, Functional Annotation, and Label-Free Quantification

The LC-MS/MS raw data were processed into mzXML files and were further searched against the Uniprot database restricted to *T. aestivum* (release May 2017, 136,892 accessions) and a contaminant database including human keratin and trypsin by using the X!Tandem pipeline software (version 3.4.3 “Elastine durcie”, http://pappso.inra.fr/bioinfo/xtandempipeline/), which encapsulates the X!Tandem program and supplies a graphical interface to manage results [73].

Enzymatic cleavage was declared as a tryptic digestion with one possible miscleavage event. The carbamidomethylation of Cys residues, as well as the possible oxidation of Met residues, were considered in the modification parameters of the software. Precursor mass and fragment mass tolerance were set at 5 ppm and 0.5 Da, respectively. The results obtained for the peptide identification were validated by filtering the peptides with an e-value below 10^−2^. Proteins were identified using at least two specific peptides and an e-value score of 10^−4^.

The *ProtAnnDB* pipeline (http://www.polebio.lrsv.ups-tlse.fr/ProtAnnDB/) was used to annotate the identified proteins [35]. To predict the subcellular localization of the proteins, three software programs were used: TargetP and SignalP, which are both included in the pipeline, and Phobius (http://phobius.sbc.su.se/). Proteins were considered to be secreted when they had no ER retention signal and displayed a predicted signal peptide from their sequence by at least two out of the three software programs. In order to identify CWPs and annotate the dataset, three functional domains prediction software programs were used: Pfam, InterPro, and Prosite, which are all included in *ProtAnnDB*. The information provided by these tools allowed for the identification of CWPs in our dataset and classified them into nine functional classes. The proteomic data of the present work was included in the *Pride* database (http://www.proteomexchange.org/; dataset identifier: PXD016258).

Spectra with confident identifications were used for label-free quantification based on extracted ion chromatograms (XICs) computed using the MassChroQ tool (MassChroQ Gui, version 2.2.2) [74]. The resulting table of peptide XICs was then processed by the Rstudio statistical environment (version 1.0.143) (Boston, MA, USA). We first applied a filter to the outlying retention times of identified peptides to prevent possible false identifications. In this scope, the standard deviation of retention times for each of them was computed through the whole dataset and a 25 s threshold was applied. In a second step, we normalized the computed XICs on runs twice to remove any engine effect with the help of the experimental TIC, and on biological replicates in order to compare lanes. More precisely, we applied a simple correction factor on each computed XIC to place 1D lanes in the same range of values. The third step filtered the peptide population. Indeed, we chose to keep only peptides that were specific to one protein, assuming that the abundances of peptides should be correlated to the abundance of the related protein. Additionally, these peptides had to be identified in two of three biological replicates within at least one condition (developmental stages or grain parts). Finally, the relative abundance of a protein in a biological replicate was computed by summing the XIC values of the selected peptides.

### 4.5. Statistical Analysis

Statistical analysis was performed on log_10_ transformed abundance values. To manage missing values, we replaced them with a value mimicking a minimum mass spectrometer detection threshold and by picking at random such a value in a range between the minimum value of the XIC dataset and half of this minimum.

The ade4 package (version 1.7-6, http://pbil.univ-lyon1.fr/ade4/home.php?lang=eng) was used to perform PCA analysis on scaled data. In order to determine the CWPs showing a significant effect of developmental stages or grain parts on their abundance, one-way ANOVA was used. Protein abundances were considered to be significantly different for *p*-values below 0.05 and a data subset of “significant proteins” was created from this analysis.

We used this subset, restricted to CWPs to focus on, to build heat maps with the gplots package on covariance matrix (version 3.0.1) (Figure 4B, Figure S3).

### 4.6. Subcellular Localization

Total RNA was extracted from wheat grains harvested from 150 to 250 GDD using an RNA kit (Qiagen, Courtaboeuf, France). RNA samples were then treated twice with a DNase buffer and purified using the RNeasy MinElute Cleanup kit (Qiagen, Dusseldorf, Germany) by following the manufacturer’s instructions. Reverse transcription was carried out with 2 µg of total RNA, anchored oligo-dT, and the Transcriptor First Strand cDNA synthesis kit (Roche Applied Science, Mannheim, Germany). The binary vector pvKH18En6 was used to generate the constructs encoding the green fluorescent protein (GFP) C-terminal fusion of the three proteins of interest. The full length cDNAs encoding a pectin methylesterase (PME, A0A1D5V0T8), a PME inhibitor (PMEI, A0A1D6BK49), and a glycoside hydrolase belonging to family 152 (GH152, A0A1D6DD47) were amplified via PCR using specific oligonucleotide primers (Table S1). The corresponding amplicons were purified and cloned into the pvKH18En6 binary vector [75] using the XbaI and BamHI restriction sites leading to the upstream fusion to GFP. The construct Sec-mRFP was used as an aploplactic marker [76]. Transformation of *A. tumefaciens* and the transient expression of the constructs in tobacco leaf epidermal cells were performed as previously described [77]. Transformed *A. tumefaciens* cultures were resuspended in an infiltration buffer at OD_600nm_ = 0.05. Fluorescence was examined with an inverted Nikon A1 confocal laser-scanning microscope (Nikon Europe, Amsterdam, The Netherlands) at three days after infiltration. Infiltrated leaf samples were placed in a solution containing 30% glycerol 5 min before imaging with the confocal microscope to plasmolyze the cells.

### 4.7. Histochemistry

Wheat grains at the developmental stages of approximately 150 GDD and 250 GDD were fixed overnight in 3% (*w*/*v*) paraformaldehyde in a 0.1 M phosphate buffer at pH7.4, dehydrated in ethanol series, and embedded with LR-White resin as previously described [14]. Transverse semi-thin sections (1-µm thickness) of grains were cut with an ultracut (UC7, Leica, Nussloch, Germany) and stained with Toluidine Blue O (1% in 2.5% Na_2_CO_3_ for 5 min, then washed in water). Stained sections were observed using a Multizoom Macroscope (AZ100M, Nikon Europe, Amsterdam, The Netherlands) under bright-field conditions.

## 5. Conclusions

Our quantitative proteomic work provides an overview of CWPs of early-developing grains of wheat, both in the endosperm and the outer layers. It provides important knowledge for a better understanding of cell wall deposition and remodeling during grain development that appears essential to controlling cereal quality and yield. Certainly due to the different tissues that composed the outer layers, more CWPs were identified, reflecting more numerous protein activities than in the endosperm. Additionally, a higher level of accumulation of a significant number of CWPs was found in the endosperm at 250 GDD, revealing strong metabolic activities in endosperm cell walls at this developmental stage. Altogether, many CWPs could be involved in the cell wall polysaccharide remodeling, as well as in lignin and cutin formation in the outer layers. In addition, an unexpected number of proteins annotated as plant invertase/PME inhibitors were found in the endosperm. Further characterization is required to confirm their precise function during grain development. To date, this is the most complete cell wall proteome from a monocot species, and the first one to be performed on separated tissues of cereal grains.

## Supplementary Materials

Supplementary materials can be found at https://www.mdpi.com/1422-0067/21/1/239/s1. Table S1: Sequences of oligonucleotide primers used for gene amplification. Table S2: (A) Proteins extracted from cell walls of the developing wheat grain (endosperm and outer layers) at two developmental stages and predicted to be secreted (the so-called CWPs). (B) Proteins extracted from the cell walls of the developing wheat grain (endosperm and outer layers) at two developmental stages and predicted to be intracellular. Table S3: Statistical analysis performed on the quantitative data corresponding to wheat CWPs identified at two developmental stages, both in the endosperm and outer layers. S3a. CWPs differentially accumulated in different grain parts at 150 GDD. S3b. CWPs differentially accumulated in different grain parts at 250 GDD. S3c. CWPs differentially accumulated in the endosperm at 150 and 250 GDD. S3d. CWPs differentially accumulated in the outer layers at 150 and 250 GDD. Figure S1: 1DE profiles of wheat grain proteins extracted from enriched cell wall fractions. Ten micrograms of proteins from each sample (three biological replicates (1–3) of proteins extracted from the outer layers (OL) or endosperm (E) at 150 GDD and 250 GDD have been separated by 1DE and stained with Coomassie Blue. Molecular mass markers (M) are in kDa. Figure S2: Distribution into functional classes of CWPs identified in the endosperm and the outer layers at 150 GDD and 250 GDD according to their predicted function. PACs: Proteins acting on cell wall polysaccharides. Figure S3: Heat maps of relative amounts of the CWPs identified in the endosperm or in the outer layers, and at one of two early developmental stages. En = endosperm samples; OL = outer layers samples; 150 = 150 GDD samples; 250 = 250 GDD samples.
