# Correction: Cherkaoui, M., Lollier, V., Geairon, A., Bouder, A., Larré, C., Rogniaux, H., Jamet, E., Guillon, F. and Francin-Allami, M. Cell Wall Proteome of Wheat Grain Endosperm and Outer Layers at Two Key Stages of Early Development. *Int. J. Mol. Sci.* 2020, *21*, 239

**DOI:** 10.3390/ijms21051740

**Published:** 2020-03-03

**Authors:** Mehdi Cherkaoui, Virginie Lollier, Audrey Geairon, Axelle Bouder, Colette Larré, Hélène Rogniaux, Elisabeth Jamet, Fabienne Guillon, Mathilde Francin-Allami

**Affiliations:** 1 INRAE, UR BIA, F-44316 Nantes, France; mehdicherkaoui04@gmail.com (M.C.); virginie.lollier@inra.fr (V.L.); audrey.geairon@inra.fr (A.G.); axelle.bouder@inra.fr (A.B.); colette.larre@inra.fr (C.L.); helene.rogniaux@inra.fr (H.R.); fabienne.guillon@inra.fr (F.G.); 2 Laboratoire de Recherche en Sciences Végétales, Université de Toulouse, CNRS, UPS, 31326 Castanet Tolosan, France; jamet@lrsv.ups-tlse.fr

The authors wish to make the following corrections to this paper [1]:

Change in Author Names (Spelling)

The authors wish to make the following correction to this paper [1]: the author name “Cherkaoui Mehdi” should be “Mehdi Cherkaoui”, “Lollier Virginie” should be “Virginie Lollier”, “Geairon Audrey” should be “Audrey Geairon”, “Bouder Axelle” should be “Axelle Bouder”, “Larré Colette” should be “Colette Larré”, “Rogniaux Hélène” should be “Hélène Rogniaux”, “Jamet Elisabeth” should be “Elisabeth Jamet”, “Guillon Fabienne” should be “Fabienne Guillon” and “Francin-Allami Mathilde” should be “Mathilde Francin-Allami” 

We apologize for any inconvenience caused to the readers.
