# Supplementary material for: Cell Wall Proteome of Wheat Grain Endosperm and Outer Layers at Two Key Stages of Early Development
Source: Int J Mol Sci. 2019 Dec 29;21(1):239. doi: 10.3390/ijms21010239 (PMC6981528; doi:10.3390/ijms21010239)
Supplement: Supplementary file 1 [file ijms-21-00239-s001.zip › Supplementary MaterialsV2.pdf]

Table S1 : Sequences of the oligonucleotides used for gene amplification

Gene accession	Forward Primer (5' → 3')	Reverse Primer (5' → 3')
A0A1D6BK49	GCTCTAGAATGAAG ATCATTCTCCTCCTC	GCGTCGACTTTAAT AACAACTTGCCAAG
A0A1D5V0T8	GCTCTAGAATGAAG TCCACCGCG	GAGTCGACTTGCCT CTCTTCTTCTC
A0A1D6DD47	GCTCTAGAATGGCT TCCCCTAAGCTT	TAGTCGACTTGTGG CAGAAGATGACCTT

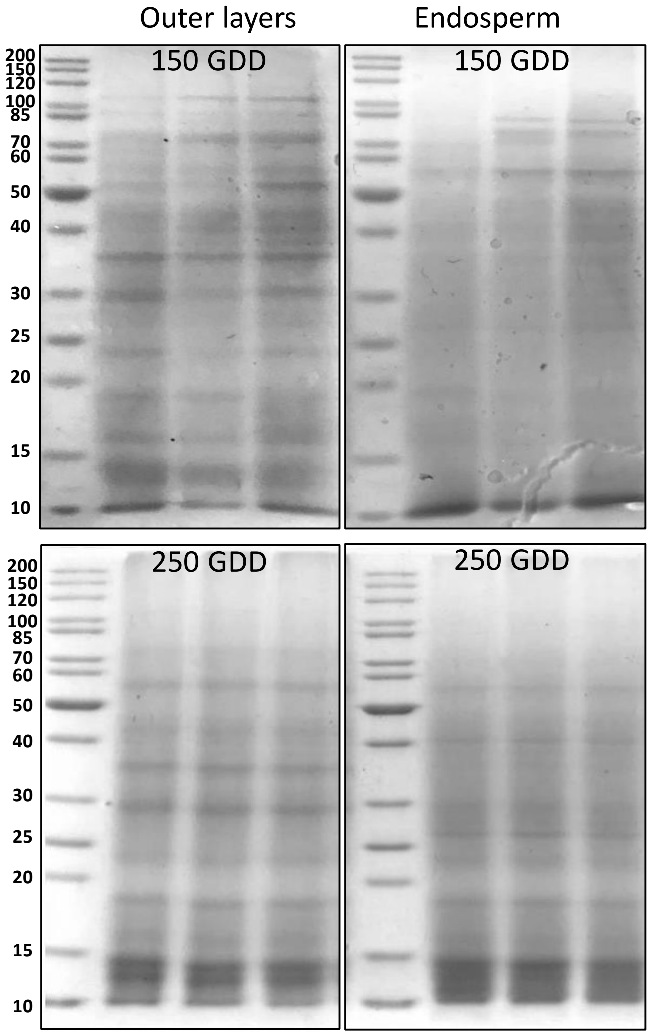

Fig. S1 1DE profiles of wheat grain proteins extracted from enriched cell wall fractions. Ten µg of total proteins from each sample (3 biological replicates of proteins extracted from outer layers or endosperm at 150 GDD and 250 GDD) have been separated by 1DE and stained with Coomassie Blue.; Molecular mass markers (M) are in kDa.

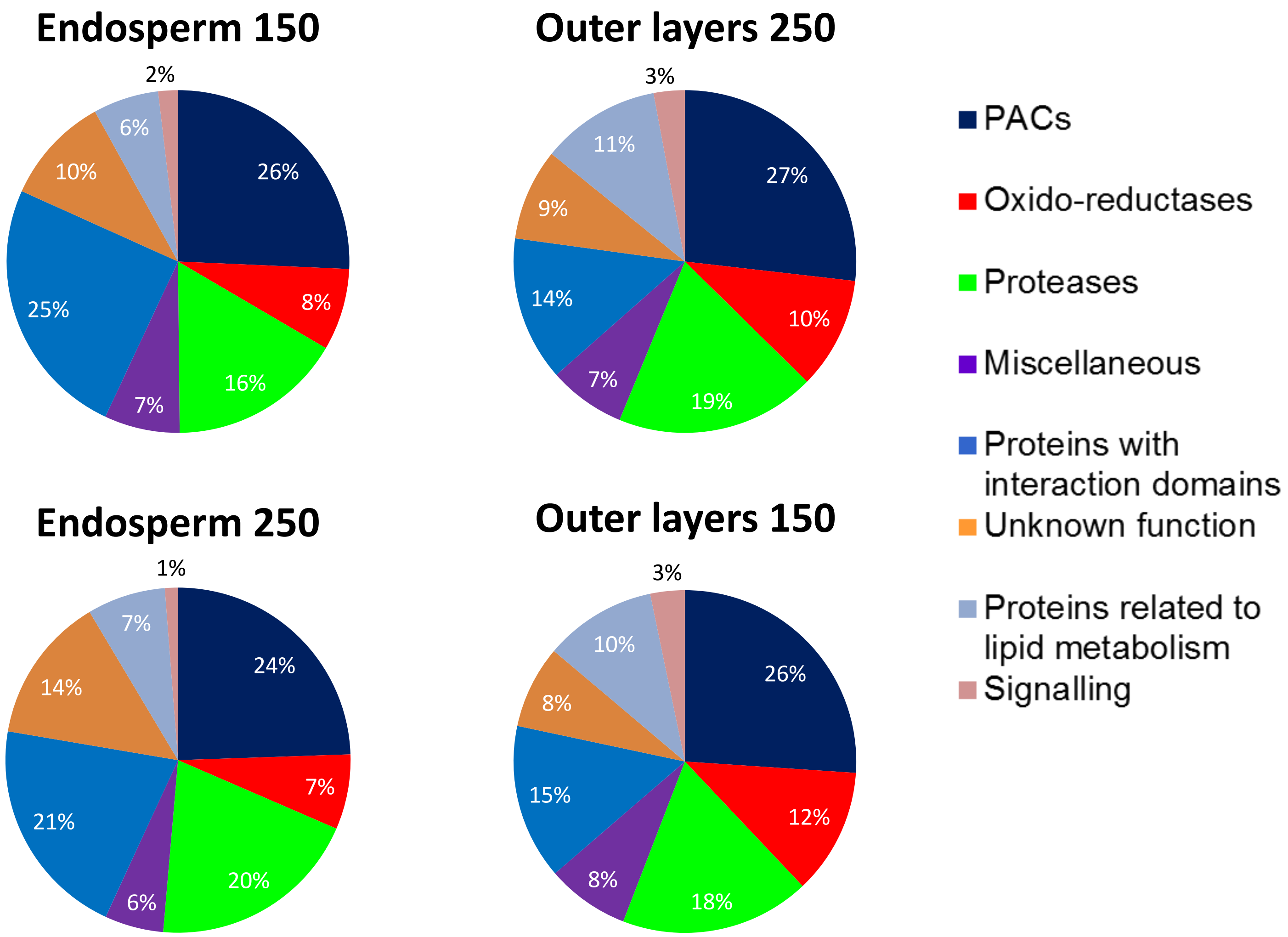

Fig. S2 Distribution into functional classes of CWPs identified in the endosperm and the outer layers at 150 GDD and 250 GDD according to their predicted function. **PACs**: Proteins acting on cell wall polysaccharides.

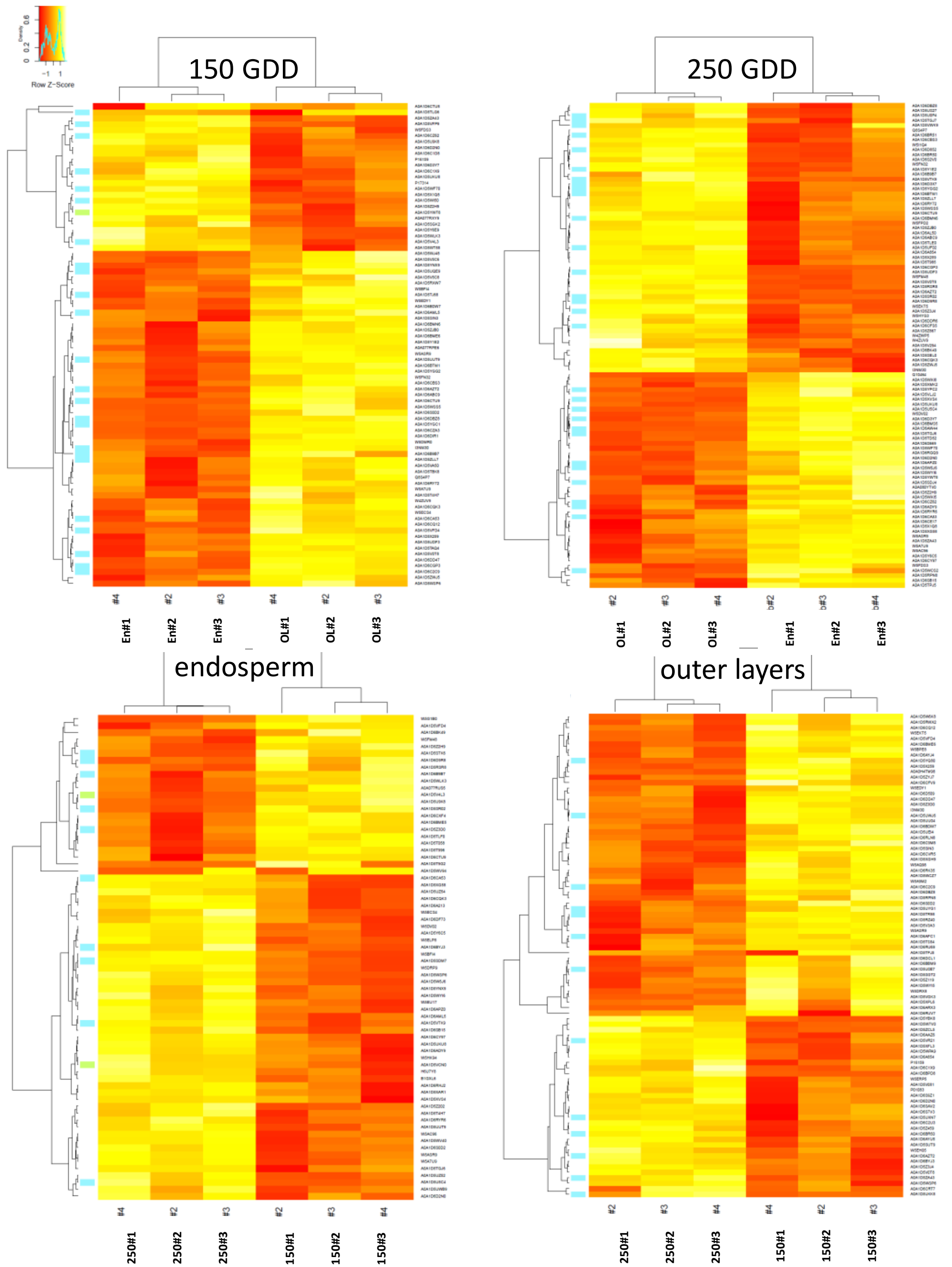

Fig. S3. Heat maps of relative amounts of the CWPs identified in the endosperm or in the outer layers, and at one of two early developmental stages. En=endosperm samples ; OL=outer layers samples ; 150=150 GDD samples ; 250=250 GDD samples.
